# A Ten-Year Perspective on Twist-Bend Nematic Materials

**DOI:** 10.3390/molecules27092689

**Published:** 2022-04-21

**Authors:** Richard J. Mandle

**Affiliations:** 1 School of Physics and Astronomy, University of Leeds, Leeds LS2 9JT, UK; r.mandle@leeds.ac.uk; 2 School of Chemistry, University of Leeds, Leeds LS2 9JT, UK

**Keywords:** liquid crystals, twist-bend nematic, liquid crystal dimers, nematic

## Abstract

The discovery of the twist-bend nematic phase (N_TB_) is a milestone within the field of liquid crystals. The N_TB_ phase has a helical structure, with a repeat length of a few nanometres, and is therefore chiral, even when formed by achiral molecules. The discovery and rush to understand the rich physics of the N_TB_ phase has provided a fresh impetus to the design and characterisation of dimeric and oligomeric liquid crystalline materials. Now, ten years after the discovery of the N_TB_ phase, we review developments in this area, focusing on how molecular features relate to the incidence of this phase, noting the progression from simple symmetrical dimeric materials towards complex oligomers, non-covalently bonded supramolecular systems.

## 1. Introduction

The term “liquid crystal” refers to a large number of states of matter that possess some degree of positional and/or orientational order, which is intermediate between isotropic liquids and crystalline solids. Much has been written about nematic liquid crystals and the twist-bend nematic phase, and so for the sake of brevity a short introduction to the topic suffices. The uniaxial nematic phase is arguably the simplest liquid crystal phase, with the constituent molecules (or particles) being, on average, oriented along a vector termed the director. Nematic liquid crystals are of special interest due to their role in display technology, and the discovery of new nematic ground states such as the twist-bend nematic is met with great enthusiasm. Biaxial nematics, in which the molecules are oriented along two orthogonal directors [1], are known to exist [2], but are outside the scope of this review. Similarly, although beyond the scope of this review, we note that nematic phases are almost exclusively *apolar*, that is, molecules orient both parallel and antiparallel to the director; very recently, the polar ferroelectric nematic phase has been shown to exist [3,4,5,6,7].

Introduction of chirality to a nematic liquid crystal leads to the formation of a chiral nematic phase, which has a helical superstructure. Dozov and Meyer independently suggested that bent shaped molecules could spontaneously form a heliconical nematic structure that is locally chiral, even when formed of achiral molecules [8]. This is termed the twist-bend nematic (N_TB_) phase, and was reported experimentally in a landmark work in 2011 [9]. The N_TB_ phase has been described as the “structural link” between the uniaxial nematic phase and the helical chiral nematic mesophase [10]. A number of techniques have measured [10,11,12,13] (or inferred [14,15]) the repeat length of the N_TB_ phase, with in situ resonant X-ray scattering being particularly noteworthy [16,17], and while the precise pitch length is material dependent, a value of around 10 nm is typical. This being said, other models have been proposed that merit further experimental investigation [18], but were outside the scope of this review.

The average conical angle between the mesogens and the helical axis can be measured by NMR [19] or birefringence [20], or by reconstructing the ODF using order parameter data from, for example, SAXS [21], polarised Raman spectroscopy [22], or NMR [23]. The conical angle of the N_TB_ phase remains below the magic angle and the phase is uniaxial with positive birefringence, confirmed by conoscopic investigation [24]. Calorimetric studies show the N_TB_-N is typically first order, and close to tricritical [9,25,26,27,28]. The N_TB_ phase has been shown to be strongly shear thinning; for the material KA(0.2), a 6-component mixture with a pitch length of 10.5 nm [29,30], it was shown that at low (<1 Pa) shear stress, the viscosity was ~1000× larger than the nematic phase. For the same material at high shear stress (>10 Pa), the viscosity of the N_TB-_phase dropped by two orders of magnitude as the helix underwent shear-induced realignment [31].

**Figure 1:**
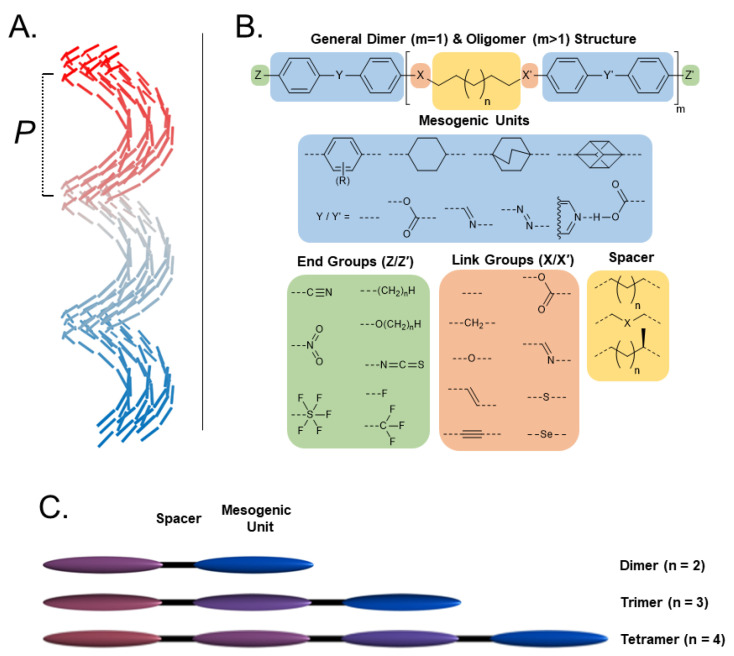
(**A**) Cartoon depiction of the heliconical director precession in the twist-bend nematic phase; mesogenic units are shown as cylinders, and are coloured according to their position along the helix axis. (**B**) The general structure of terminally appended liquid crystalline dimers and oligomers, subdivided into regions of interest to this review. For each subdivision example, chemical fragments that have been utilised in N_TB_ materials are given. (**C**) Schematic depiction of the relationship between dimers, trimers, and tetramers in terms of their subunit composition.

As with all mesophases, the formation of the N_TB_ phase in a given material is intimately linked to molecular structure, and there has been significant effort in the design of new materials that exhibit the N_TB_ phase [32]. The molecular structure of liquid crystalline dimers can be subdivided into distinct regions, as outlined in Figure 1B. In the simplest terms, a dimer consists of two rigid mesogenic units joined by a flexible spacer [33,34,35]. For a trimer, three mesogenic units are joined in a similar fashion, and so on. Today, in the region of 1000, materials are known to exhibit the N_TB_ phase, and so in this review, we focused on systematic variations to key areas of molecular structure rather than making a futile attempt to cover all materials.

## 2. Materials

The CBnCB family are archetypal N_TB_ materials, and a logical place to begin our review. These materials feature two cyanobiphenyl mesogenic units separated by *n* methylene units. As with all LC dimers, the CBnCB family displays a strong odd–even effect, with the even parity members displaying notably higher clearing points than those with odd spacer parity. With the exception of the shortest homologue (CB3CB), all odd parity CBnCBs displayed two nematic phases, the lower temperature nematic phase being identified as the N_TB_ phase (Table 1). Even parity CB*n*CB materials displayed only a conventional nematic phase.

Due to their favourable working temperatures, the properties of the N_TB_ phase of some members of the CB*n*CB family have been quite well explored. The helix pitch length of CB7CB was measured by freeze-fracture TEM by Chen et al. [11], who found a value of 8.3 nm. Later, Zhu et al. measured the N_TB_ pitch length of CB9CB as a function of temperature by resonant X-ray scattering at the carbon K-edge [16]; the pitch length was largest close to the N_TB_–N transition (~9.8 nm), and decreased to around 8 nm with decreasing temperature. Yu and Wilson recently reported fully atomistic MD simulations of CB7CB, which yielded an N_TB_ phase with a pitch length of 8.35 nm [41]. The conical tilt-angle within these simulations (~29°) agreed well with the experimental values [11,20].

The orientational order parameters of several members of the CB*n*CB family have been measured, with results from different methods generally being consistent with one another. For odd parity CB*n*CBs, the thermal evolution of orientational order within the nematic phase is unremarkable, however, at T_N_TB_-N_, there is a decrease in orientational ordering. This decrease results from the molecules tilting away from the helix, which manifests as a reduction in, or even negative value of <*P*4> [21,42]. This behaviour of the orientational order parameters has also been observed by NMR [43], and is reported to be consistent with the polar twisted nematic (N_PT_) model of the N_TB_ phase. CB8CB, which has even spacer parity and does not show the N_TB_ phase, displays unusually large nematic order parameters [22], as does CB10CB [43].

We next consider variations in the mesogenic units. Compound **11** belongs to a class of materials known as “PZP” dimers (P = phenyl, Z = carboxylate ester). The synthesis of these materials is trivial; the penultimate step is esterification of *bis* 1,9-(4-hydroxyphenyl)nonane, permitting the synthesis of a large number of variations in core structure via esterification. In Table 2, we present the transition temperatures of a set of PZP-9-PZP dimers with varying terminal groups. While cyano, isothiocyanato, and alkyl/alkoxy groups are found to support the formation of the N_TB_ phase [44,45], various other polar units (nitro, fluoro, trifluoromethyl, pentafluorosulphanyl) render the resulting materials non-mesogenic [44]. For compound **15**, the –NCS unit enables measurement of the N_TB_ pitch length (~9 nm) using resonant X-ray scattering at the carbon K-edge and also the sulphur K-edge [46].

The synthetic flexibility afforded by the PZP dimers makes it possible to prepare dissymmetric materials such as those shown in Table 3 [47,48]. With a single phenyl 4-cyanobenzoate mesogenic unit, it is possible to obtain materials that display the N_TB_ phase, even when the second mesogenic unit incorporates an ‘unfavourable’ terminal unit (e.g., NO_2_, SF_5_, etc.) [47,48]. This approach also lends itself to the synthesis of trimers, tetramers, and so on, as will be discussed later. A general trend in the materials presented in Table 3 is that the addition of additional fluorine atoms *ortho* to the terminal group leads to depressions in both T_N_TB_-N_ and T_N-Iso_, mirroring the behaviour of calamitic materials [49]. One advantage enjoyed by unsymmetrical materials is that their melting points are generally lower than those of the corresponding symmetrical derivatives, which compensates for the more elaborate synthesis required.

Turning now to variations in the linking groups and spacer regions, while the N_TB_ phase is most commonly associated with dimers incorporating a methylene spacer, it is also found in a large number of materials with imine linking units. The imine-linked (3-hydroxylphenyl) 4-alkylbenzoate dimers reported by Šepelj et al. (Table 4) showed a delicate balance between columnar, nematic, and N_TB_ mesophases, with the specific phase type being dependent upon both the length of the central spacer as well as the peripheral alkyl chains [50].

Analogous in structure to the materials shown in Table 5, Šepelj et al. reported a family of imine linked phenyl 4-alkoxybenzoate dimers. Only one member displayed the twist-bend nematic phase, with *m* = 7 and *n* = 4, the majority of the materials displaying a B6 type mesophase. 

We now explore the role of the chemical makeup of the central spacer beyond the methylene and imine systems already discussed. Archbold et al. reported a family of cyanobiphenyl dimers that are homologous to CB7CB in structure, having spacers of comparable length (seven methylene or equivalent units) but with different chemical makeup (Table 6) [54]. Materials incorporating two alkyne units were non-mesogenic. The onset temperature of the N_TB_ phase was significantly reduced for the dipropyl ether spacer (**55**), while the N_TB_ phase was absent for **61**, which included a diethylegylcol spacer. The *bis* imine material **60** exhibited a direct N_TB_ to isotropic transition temperature. Archbold et al. linked the observed transition temperatures to the average bend of the molecule, itself obtained as a probability weighted average of many conformers obtained with the rotational isomeric state (RIS) approximation, with the suggestion that an ‘optimal’ bend angle exists for the N_TB_ phase, which leads to, *inter ali*a, direct N_TB_–Iso transitions. This is exemplified by the high thermal stabilities of the N_TB_ phases of the ketone-linked material (CBK-5-KCB) as well as the imine-linked material (CBI-3-ICB).

Refining the earlier approach of Archbold et al., Mandle and Goodby investigated the conformational preference of a series of homologues of CB9CB (Table 7) [55,56]. Again, the rotational isomeric state approximation was used to generate conformational libraries for each material; the average bend angle between the two mesogenic units then being calculated as a probability weighted average. Conformational ensembles were validated by comparison of average inter-proton distances with those obtained from ^1^H-^1^H NOESY NMR experiments. It is suggested that the stability of the N_TB_ phase is related to the average bend angle, specifically, a high ratio of T_N_TB_-N_ to T_N-Iso_ is achieved by having an average bend angle in excess of 110°.

We now consider four families of related cyanobiphenyl dimers with varying linking groups and spacer lengths (Table 8) [59,60,61]. The chemical makeup of each family is evident from their names (e.g., the CB*n*OCB series feature one methylene and one ether linking unit, the CB*n*SCB series feature one methylene and one thioether linking unit, and so on). Further examples from each family are to be found in the given references. Generally, the transition temperatures of thioether containing materials are lower than the equivalent CB*n*OCB material, with the difference being most pronounced for shorter spacer lengths. A simple explanation suffices here, with a deeper understanding needing to draw on other relevant conformational effects. Consider that a methylene unit imparts a tetrahedral bond angle (109.5°), an ether gives an angle of 104.5°, but an arylthioether has a bond angle of ~90°. The influence of thioethers is therefore most pronounced for shorter chain lengths, whereas for longer spacers, they are somewhat offset. The thioether is somewhat unfavourable for the formation of the N_TB_ phase as it tends to depress the average bend angle away from the apparent favoured value of >110 °C. However, for some materials (CBO7SCB, **67**, and CBS7SCB, **66**) the melting point is suppressed to such a degree that the materials are in the N_TB_ phase at ambient temperature, which is a remarkable achievement that greatly simplifies experimentation. The helical pitch length of several compounds in Table 8 has been reported: CB6OCB ~10–15 nm [62], CBS7SCB 8.7 nm, CBO7SCB 18.4 nm, and CBO5SCB 14.8 nm [63]. Linking the N_TB_ pitch length to molecular geometric parameters appears to be a logical future direction. Almost simultaneously with Arakawa et al. [61], the CBS*n*SCB and CBO*n*SCB materials were also synthesised and reported independently by Imrie et al. [64]. Imrie et al. also reported the pitch lengths of CBO5SCB (~8.9 nm), CBS7SB (~8.7 nm), and the mixed cyanoterphenyl/cyanobiphenyl material, CT6SCB (~9.7 nm); in all three cases, this corresponded to approximately four end-to-end molecular lengths. 

Arakawa et al. subsequently demonstrated cyanobiphenyl dimers with mixed thioether/ketone linking units [65] (Table 9). Earlier, Archbold et al. found that ketone-linking units generated highly stable N_TB_ phases due to their favourable bend angles (Table 6) [54]. Employing mixed ketone and thioether units showed a dramatic increase in the N_TB_ onset temperature when compared to the equivalent materials employing either two thioethers (CBS*n*SCB), or one thioether and one other linking unit (CB*n*SCB, CBO*n*SCB, CBS*n*SCB). Again, a simple explanation suffices for the sake of this review: the ketone unit has a bond angle of ~120°, which offsets the unfavourable angle imposed by the thioether. Clearly, a detailed DFT study of the conformational landscape of these materials (and indeed, others) appears warranted, and presents one possible route to further understanding these intriguing materials. 

## 3. Chiral N_TB_ Materials

The N_TB_ phase has been described as the “structural link” between the conventional nematic phase and the helical chiral nematic (cholesteric) phase [10]. As discussed, the N_TB_ phase has a helical structure and when formed from achiral molecules, there is no preference for left- or right-handed helices. Our focus in this review was on molecular structure and the materials that generate the N_TB_ phase, so our focus was on examples whereby chirality results from the molecular structure of the dimer itself, rather than systems in which chirality is introduced via an additive [66].

Gorecka et al. reported a number of ester-linked unsymmetrical dimers that incorporated cholesterol as a mesogenic unit; these materials are chiral, and thus they are the first reported chiral N_TB_ materials. The length of the central spacer and its parity dictate the balance between exhibiting N_TB_ (odd parity) or SmA (even parity) mesophases in these materials. The mesophase behaviour of these materials is more complex than shown in Table 10, with the materials exhibiting multiple nematic phases and/or blue phases. The N_TB_ pitch length of **90** was measured to be 50 nm by in situ AFM, notably larger than that of CB7CB, but still about a few molecular lengths [24,67]. Later, the helical pitch length of **99** was measured by resonant carbon K-edge X-ray scattering and found to take a value of ~11 nm [68]. The pitch length of the chiral nematic phase was determined to be 224 nm by the same method. 

Gorecka et al. also reported another family of cholesterol containing dimers that exhibited the N_TB_ phase (Table 11), with some members also exhibiting a smectic phase of unknown structure (SmX) [24]. The pitch length of **104** in the N_TB_ phase was measured by the resonant X-ray scattering method, with a temperature dependent value of 13.3–20.3 nm. Conversely, the chiral nematic pitch length for this material was measured by the same technique to be 220 nm [68]. Although not reported upon, the incorporation of an azo unit presumably enables isothermal N_TB_ transitions in these materials (*via* photoisomerisation), as first reported by Paterson et al. [69]. 

Mandle and Goodby reported the synthesis of symmetrical LC dimers in which the central spacer is itself chiral (Table 12); starting from (R)-2-methylglutaric acid, four synthetic steps telescoped into two reactions afforded the key (R)-bis-1,5-(4-hydroxyphenyl)-2-methylpentane intermediate, which was elaborated using standard esterification protocols, affording compounds **107**–**113**. The measured helical twisting power of **107** was rather low (0.36 mm^−1^ wt%^−1^) due to the large degree of conformational freedom experienced by the lateral methyl unit. The N_TB_ phase was only exhibited by materials whose mesogenic units had a large aspect ratio due to the unfavourable conformational effects of the lateral methyl unit within the central spacer. However, this also gave rise to the unusual SmA–N_TB_ transition in compounds **111** and **112**, which has previously only been observed for a small handful of materials [70].

Walker et al. reported a family of unsymmetrical dimers that are terminated by either butyl, racemic 2-methylbutyl, or (S)-2-methylbutyl chains (Table 13) [71]. The N_TB_–N transition temperature was marginally higher for chiral materials than the achiral analogues, in agreement with the theoretical predictions [72]. Notably, the N_TB_ phase formed by **119** was found to be miscible with that of the achiral material CB6OCB (**77**). 

## 4. Bent-Core Systems

Compared to dimers comprising rod-like (calamitic) mesogenic units, there are relatively few examples of bent-core liquid crystals that exhibit the N_TB_ phase. A family of no symmetrical bent-core materials with a central phenyl piperazine group were prepared by Schroder et al. [73] with shorter homologues exhibiting two nematic phases, denoted as N_X_. Longer chain homologues exhibited the SmC_P_ phase. Later, compound **126** was studied by FFTEM, and the lower temperature nematic was shown to be a N_TB_ phase with a pitch length of 14 nm—larger than CB7CB, but of the order of a few molecular lengths [12]. Based on this, it seems probable that the ‘N_X_’ phase of compounds **124** and **125** is also the N_TB_ phase. 

**Figure 2:**
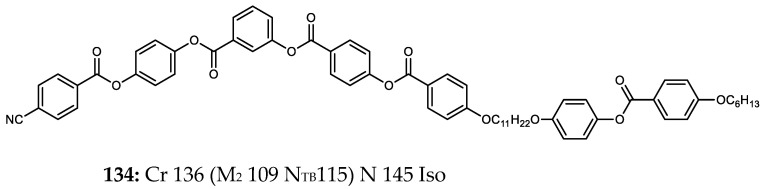
Chemical structure and transition temperatures (°C) of the hybrid bent-core/calamitic dimer reported by Tamba et al. [74]. Phase transitions are presented in parenthesis are monotropic.

Tamba et al. reported an ether-linked dimer, comprising a bent-core unit as well as a calamitic unit, which exhibited the twist-bend nematic phase as well as an unidentified ‘M_2_’ mesophase [74]. Homologues employing other spacer lengths (trimethyleneoxy or hexamethyleneoxy) or a dodecyloxy terminal chain *in lieu* of the nitrile unit employed in **134** do not exhibit the twist-bend nematic phase. To date, compound **134** (Figure 2), along with those in Table 14, are the only known examples of bent-core materials that exhibit the twist-bend nematic phase, although others have been previously suggested [75].

## 5. Beyond Dimers: Trimers, Tetramers and Oligomers

Now, our focus shifts beyond covalent dimers to equivalent trimers, tetramers, and so on [76,77,78,79,80,81,82,83,84,85]. We will focus first on materials of special note before considering the behaviour of families of materials. CB6OBA, a hydrogen bonded LC-trimer, was the first oligomeric material shown to exhibit the N_TB_ phase (Table 15) [86]. The hexamethyleneoxy spacer of CB6OBA imparts a gross bent shape to the hydrogen bonded dimer, permitting the formation of the N_TB_ phase, which is absent for the even parity homologue, CB5OBA. The terminal carboxylic acid group contains a hydrogen bond donor and acceptor, thus, both open and closed forms can be observed [87,88].

**Figure 3:**
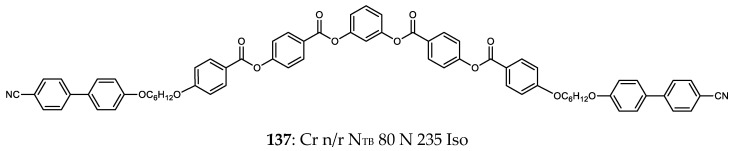
Chemical structure and transition temperatures (°C) of the hybrid bent-core/calamitic trimer (**137**) reported by Wang et al. [13]. The melting point was not reported.

Wang et al. reported a symmetrical trimer that features two cyanobiphenyl units appended to a bent-core [13]; curiously, spacers in this system have even parity, with the requisite bent shape resulting from the 1,3-disubstituted phenyl ring employed within the bent-core unit. The pitch length of the N_TB_ phase of **137** (Figure 3) was measured to be 19 nm using the FFTEM method, which was roughly four molecular lengths. X-ray scattering showed that the nematic phase is intercalated, with the d-spacing of the diffuse small angle peak being at ~1/3 of the molecular length. The orientational order parameters <*P*2> and <*P*4> were measured by X-ray scattering, both being found to decrease on entering the N_TB_ phase. Replacement of a single cyanobiphenyl with a decyloxy chain was shown to eliminate the N_TB_ phase [89].

**Figure 4:**
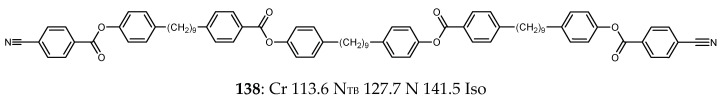
Chemical structure and transition temperatures (°C) of the linear tetramer reported by Mandle and Goodby.

In 2016, Mandle and Goodby reported a methylene linked tetramer comprising phenyl benzoate mesogenic units (Figure 4) [47,90]. The heliconical tilt angle of the N_TB_ phase of **138** was estimated via the X-ray scattering method, and was found to be comparable to the parent dimer, compound **11** (Table 2) [42]. The synthetic strategy used to prepare this tetramer was further refined to deliver a twist-bend nematic hexamer, with six mesogenic units connected in a linear manner. 

**Figure 5:**
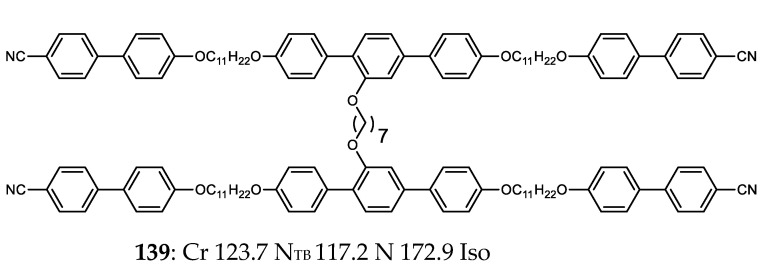
Chemical structure and transition temperatures (°C) of the non-linear hexamer reported by Mandle and Goodby.

So far, all of the materials encountered have had a linear sequence of mesogenic units. The hexamer **139** features two trimers appended via a central heptamethylenedioxy spacer (Figure 5) [91]. The individual trimers are themselves mesogenic and display the N_TB_ phase; however, the N_TB_–N transition occurs at a significantly higher temperature in the duplexed hexamer [91].

Jákli et al. reported a homologous family of 2′,3′-difluoroterphenyls [92], from the simple monomer (*n* = 0, **140**, Table 16) to the homologous tetramer **143**. Some homologous dimers, with varying terminal/spacer chain length, have also been reported [93,94,95], and a wealth of investigations have been performed on this family of materials [96,97,98].

The monomeric material displays only a nematic phase whereas those with two or more mesogenic units display nematic and N_TB_ phases, with the transition temperature increasing as the number of mesogenic units is increased. So far, we have considered odd–even effects as being restricted to those resulting from the parity of the central spacer. However, for the ‘DTC5-C9’ family, Jákli et al. showed remarkable odd–even effects in birefringence, bend elastic constants, and X-ray scattering, which resulted from the number of mesogenic units [92].

Arakawa et al. reported two families of symmetrical liquid crystalline trimers featuring ether (CBOnOBOnOCB, Table 17) [99] and mixed ether/thioether (CBSnOBOnSCB, Table 18) [100] linking groups, but with varying spacer lengths. For a given chain length, the former family exhibited higher transition temperatures than the latter. In both families, materials of even parity displayed nematic and smectic A phases, whereas those of odd parity showed nematic and N_TB_ phases.

Al-Janabi and Mandle reported a set of liquid crystalline trimers that incorporated various saturated hydrocarbon rings, isosteric with 1,4-disubstituted benzene (Table 19) [101]. Only the 2,6-cuneane material did not exhibit the N_TB_ phase, and this was attributed to the unfavourable bend angles imposed by this non-linear motif. For the materials that exhibited the N_TB_ phase (X = 1,4-benzene, 1,4-cyclohexane, and 1,4-cubane), the heliconical tilt angle was found to be effectively independent of the chemical makeup of the central ring.

The relationship between the stability of the N_TB_ phase and oligomer shape was studied in detail for a family of cyanobiphenyl/benzylideneaniline oligomers (Table 20) [102]. Due to the explored variations in the composition and parity of the spacer units, neighbouring mesogenic units within this family can have both ‘bent’ or ‘linear’ configurations. While all materials exhibit the N_TB_ phase, the heliconical pitch length has a strong dependence on the gross molecular shape; the all bent material **166** has three odd-parity spacers and a pitch of 7 nm, the bent-linear-bent tetramer **170** has a pitch of 12 nm, and the linear-bent-linear tetramer **172** has a pitch length of ~17 nm. 

## 6. Supramolecular N_TB_ Materials

We now consider supramolecular LC dimers that result from non-covalent bonds, both achiral and chiral, which exhibit the N_TB_ phase. The hydrogen bonded-LC trimer *CB6OBA* was the first reported supramolecular N_TB_ material, however, as the complex incorporates two identical molecules, there is no scope for tunability of structure. Walker et al. demonstrated a remarkable pair of supramolecular LC dimers that incorporates dissimilar hydrogen bond acceptors and donors. The parent 4-methoxybiphenyl/stilbazole dimer (**173**) is non-mesogenic; complexation with either 4-butoxy- or 4-pentyloxybenzoic acid affords the isolable supramolecular complexes shown in Table 21, both of which exhibit the N_TB_ phase [103]. While many materials are known to exhibit transitions from the N_TB_ phase to tilted (SmC, *vide infra*) or heliconical (SmC_TB_) [104] phases, complex **175** is unusual in that it exhibits a transition from the N_TB_ phase to an orthogonal smectic A phase, as do compounds **111** and **112**.

This design strategy was also used to deliver chiral supramolecular N_TB_ materials by use of the cyanobiphenyl/stilbazole system with an appropriate chiral benzoic acid [105]. The transition temperatures for this chiral supramolecular complex are somewhat lower than those of the linear analogue (shown in Table 22), and this depression of transition temperatures by branched alkyl chains is a general phenomenon in LC dimers. Resonant soft X-ray scattering at the carbon K-edge was used to measure the pitch length of **176**, which was found to take a temperature dependent value of 8.1–8.4 nm, or around two complex lengths. 

Walker et al. subsequently demonstrated the CB6OCB:*n*OS series of materials, utilising the benzoic acid/stilbazole system [106]. A weak odd–even effect was seen for both the N_TB_–N and N–Iso transition temperatures. Homologues with longer terminal chain length (*n* ≥ 4) also exhibited smectic C phases. For homologues with longer chains still (*n* ≥ 8, not shown in Table 23), the N_TB_ phase was absent, instead, the materials showed SmA and SmA_B_ phases [106].

In the same paper, further elaboration of the cyanobiphenyl/stilbazole system to unsymmetrical supramolecular trimers comprising CB6OBA and a stilbazole dimer was reported (Table 24). Although the two materials differed in their melting point, the N_TB_–N and N–Iso transitions were only slightly different. We note that, although examples are presently limited to hydrogen bonded systems, there is no obvious reason why other types of non-covalent interactions could not be employed in the design of N_TB_ materials (e.g., halogen bonds [107]), and presents a logical avenue for future research.

## 7. Summary and Outlook

The decade since the experimental discovery of the N_TB_ phase by Cestari et al. has seen a resurgence of interest in liquid crystalline dimers and oligomers. Recently, there has been a notable move from symmetrical methylene-linked dimers to more complex forms: chiral systems, photoresponsive dimers, supermolecular materials, higher and non-linear oligomers, and polymers. The recent development of room temperature materials greatly facilitates the exploration of the rich physics of these systems. 

General design principles for N_TB_ materials are always evolving; with the ability to tune molecular bend/shape through synthetic chemistry, the ability to prepare oligomers, and supramolecular systems, it is possible to obtain twist-bend nematic materials through rational design rather than through ad hoc experimentation. This being said, the majority of twist-bend nematic materials follow the tried-and-tested formula of end-to-end appended mesogenic units, with only a handful of bent-core materials and a single non-linear oligomer falling outside of this description. It is interesting to speculate as to whether more unconventional molecular geometries (e.g., lambda shaped trimers, mixed rod-disk architectures) are capable of supporting twist-bend nematic order.

Different materials display rather different helix pitches that themselves evolve differently over temperature; an understanding of this from a molecular perspective is currently elusive, but appears a reasonable proposition for future work. The potential for incorporating stimuli responsive groups, coupled with the remarkable physical properties of this phase of matter, suggests that interest in this area will continue for some time. 

## Figures and Tables

**Table 1:** Transition temperatures (T_A-B_, °C) of the CB*n*CB family of materials [9,23,26,36,37,38,39,40].

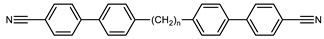					
**No.**	**Name**	***n***	**T_MP_**	**T_N_TB_-N_**	**T_N-Iso_**
**1**	CB3CB	3	142.1	-	-
**2**	CB5CB	5	150	92	97
**3**	CB6CB	6	183	-	230
**4**	CB7CB	7	102	104.5	116
**5**	CB8CB	8	175	-	195.9
**6**	CB9CB	9	83	105.0	119.8
**7**	CB10CB	10	140	-	174.1
**8**	CB11CB	11	99.9	108.6	125.5
**9**	CB12CB	12	139	-	157
**10**	CB13CB	13	106	105	122

**Table 2:** Transition temperatures (T_A-B_, °C) of some symmetric (PZP)-9 materials with varying terminal unit [44,45].

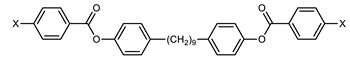				
**No.**	**X**	**T_MP_**	**T_N_TB_-N_**	**T_N-Iso_**
**11**	–CN	157.6	114.5	146.6
**12**	–NO_2_	105.4	-	-
**13**	–F	97.6	-	-
**14**	–CF_3_	102.4	-	-
**15**	–NCS	97.7	103.7	127.4
**16**	–SF_5_	123.0	-	-
**17**	–C_5_H_11_	72.8	58.8	66.2
**18**	–OC_5_H_11_	71.8	76.0	86.6

**Table 3:** Transition temperatures (T_A-B_, °C) of some dissymmetric (PZP)-9 materials with varying terminal unit [47,48].

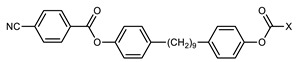				
**No.**	**X**	**T_MP_**	**T_N_TB_-N_**	**T_N-Iso_**
**11**	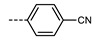	157.6	114.5	146.6
**19**	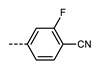	112.6	95.0	120.7
**20**	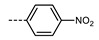	115.4	100.5	124.9
**21**	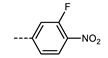	92.6	78.8	97.6
**22**	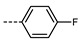	86.2	78.2	95.9
**23**	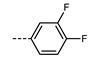	83.2	63.8	79.0
**24**	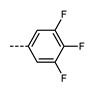	93.8	46.0	60.0
**25**	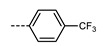	110.0	69.6	78.3
**26**	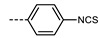	95.6	100.0	123.8
**27**	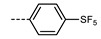	102.2	61.2	72.8
**28**	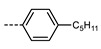	88.7	80.7	95.1
**29**	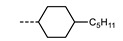	91.9	85.2	110.0

**Table 4:** Transition temperatures (T_A-B_, °C) of some imine-linked phenyl 4-alkoxybenzoate dimers [51,52,53].

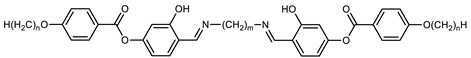						
**No.**	***n***	***m***	**T_MP_**	**T_Col-N/Iso_**	**T_N_TB_-N_**	**T_N-Iso_**
**30**	4	5	106	-	-	102
**31**	6	5	113	-	87	97
**32**	8	5	103	99	-	102
**33**	10	5	105	111	-	-
**34**	12	5	107	116	-	-
**35**	14	5	105	120	-	-
**36**	4	7	119	-	-	121
**37**	6	7	98	-	93	113
**38**	8	7	103	-	-	110
**39**	10	7	97	106	-	108
**40**	12	7	100	112	-	-
**41**	14	7	99	117	-	-

**Table 5:** Transition temperatures (T_A-B_, °C) of salicylaldimine dimers with odd spacer parity [50].

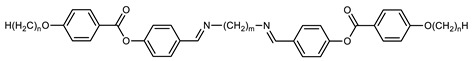						
**No.**	***n***	***m***	**T_MP_**	**T_B6-N/Iso_**	**T_N_TB_-N_**	**T_N-Iso_**
**42**	4	5	114.0	99.1	-	102.0
**43**	6	5	123.5	116.9	-	-
**44**	8	5	94.2	121.0	-	-
**45**	10	5	88.5	109.5	-	-
**46**	12	5	96.2	96.1	-	-
**47**	14	5	101.2	-	-	-
**48**	4	7	112.2	84.4	96.6	115.0
**49**	6	7	96.4	114.7	-	-
**50**	8	7	111.2	119.5	-	-
**51**	10	7	100.1	110.1	-	-
**52**	12	7	90.9	99.3	-	-

**Table 6:** Transition temperatures (T_A-B_, °C) of cyanobiphenyl derivatives with varying central spacer composition, equivalent to heptamethylene [54].

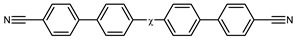						
**No.**	**Name**	**χ**	**T_MP_**	**T_N_TB_-N_**	**T_N-Iso_**	**Ave. Bend/°**
**53**	CBT3TCB	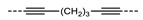	160.5	-	-	n/r
**54**	CBT1O1TCB	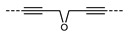	>225	-	-	n/r
**4**	CB7CB	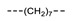	104.4	105.5	118.9	103.5
**55**	CB3O3CB	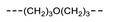	100.5	46.0	68.0	91.0
**56**	CBT4OCB	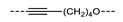	132.8	97.0	145.2	100.5
**57**	CB6OCB	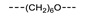	102.1	110.5	154.2	104.4
**58**	CBO5OCB	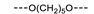	137.9	81.3	189.2	102.9
**59**	CBK5KCB	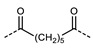	158.1	145.1	189.4	108.2
**60**	CBI3ICB	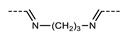	170.8	114.9	-	115.1
**61**	CBO2O2OCB	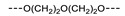	150.5	-	157.8	104.5

**Table 7:** Transition temperatures (T_A-B_, °C) of cyanobiphenyl derivatives with varying central spacer composition, equivalent to nonamethylene [54,55,56,57,58]. * Glass to N_TB_ transition.

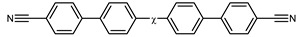						
**No.**	**Name**	**χ**	**T_MP_**	**T_N_TB_-N_**	**T_N-Iso_**	**Ave. Bend/°**
**6**	CB9CB	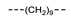	83.3	105.4	121.5	103.1
**62**	CBI7ICB	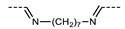	140.8	114.7	138.7	111.5
**63**	CB8KCB	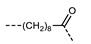	127.8	128.1	153.9	98.5
**64**	CBT6OCB	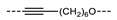	137.1	102.0	153.6	98.5
**65**	CB8OCB	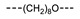	110.6	109.9	153.3	100.7
**66**	CBS7SCB	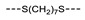	15.9 *	88.3	115.2	99.2
**67**	CBS7OCB	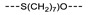	55.0	95.9	146.7	96.8
**68**	CBSe7SeCB	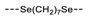	80.8	43.1	71.9	98.8
**69**	CBcZ5OCB	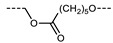	95.1	39.8	91.3	93.0
**70**	CBcO5OCB	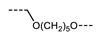	122.4	71.3	129.9	96.8
**71**	CBO7OCB	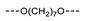	120.0	-	-	-
**72**	CBT5CBT	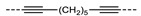	169.1	-	-	-

**Table 8:** Transition temperatures (T_A-B_, °C) of cyanobiphenyl derivatives with methylene, ether, or thioether linking groups and varying spacer length: selected members of the CB*n*OCB [59,60], CB*n*SCB [61], CBO*n*SCB [61], and CBS*n*SCB [61] series. * Glass to N_TB_ transition.

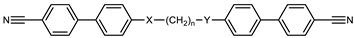							
**No.**	**Name**	***n***	**X**	**Y**	**T_MP_**	**T_N_TB_-N_**	**T_N-Iso_**
**73**	CB4OCB	3	–CH_2_–	–O–	121	103	143
**74**	CB4SCB	3	–CH_2_–	–S–	126.2	70.3	86.8
**75**	CBO3SCB	3	–O–	–S–	101.1	47	137.5
**76**	CBS3SCB	3	–S–	–S–	70.1	44.0	83.2
**77**	CB6OCB	5	–CH_2_–	–O–	99	109	155
**78**	CB6SCB	5	–CH_2_–	–S–	99.0	89.6	113.2
**79**	CBO5SCB	5	–O–	–S–	59.5	90.1	143.8
**80**	CBS5SCB	5	–S–	–S–	68.9	78.0	107.8
**65**	CB8OCB	7	–CH_2_–	–O–	112	108	154
**81**	CB8SCB	7	–CH_2_–	–S–	92.6	93.9	117.8
**67**	CBO7SCB	7	–O–	–S–	16.0 *	95.9	146.7
**66**	CBS7SCB	7	–S–	–S–	15.9 *	88.3	115.2
**82**	CB9OCB	9	–CH_2_–	–O–	116	107	148
**83**	CB9SCB	9	–CH_2_–	–S–	103.4	95.5	119.0
**84**	CBO9SCB	9	–O–	–S–	98.0	95	143.0
**85**	CBS9SCB	9	–S–	–S–	100.8	89	116.7

**Table 9:** Transition temperatures (T_A-B_, °C) of cyanobiphenyl derivatives with mixed ketone/thioether linking units [65].

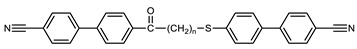					
**No.**	**Name**	***n***	**T_MP_**	**T_N_TB_-N_**	**T_N-Iso_**
**86**	CBK3SCB	3	114.6	104.6	132.6
**87**	CBK5SCB	5	120.9	110.0	152.3
**88**	CBK7SCB	7	121.8	113.6	152.1
**89**	CBK9SCB	9	155.8	-	145.0

**Table 10:** Transition temperatures (T_A-B_, °C) of unsymmetrical cholesterol containing dimers.

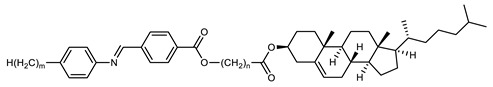						
**No.**	***n***	***m***	**T_MP_**	T_SmA-N*_	T_N_TB_-N*_	T_N*-Iso_
**90**	3	1	112.3	-	55.1	67.7
**91**	4	1	127.9	146.9	-	191.8
**92**	5	1	92.5	-	67.9	102.3
**93**	6	1	152.3	120.0	-	158.4
**94**	7	1	83.0	-	75.7	110.8
**95**	9	1	81.2	-	75.7	113.6
**96**	10	1	82.7	-	-	137.5
**97**	15	1	62.9	-	63.2	107.9
**98**	5	2	74.0	-	60.6	93.6
**99**	5	3	77.5	-	62.2	98.8
**100**	5	4	44.2	71.9	-	99.3

**Table 11:** Transition temperatures (T_A-B_, °C) of unsymmetrical dimers comprising cholesterol and azobenzene units.

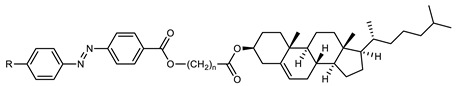						
**No.**	***n***	**R**	**T_MP_**	T_SmX-N*_	T_N_TB_-N*_	T_N*-Iso_
**101**	5	–CH_3_	83.4	-	67.1	105.5
**102**	5	–OCH_3_	84.3	-	80.8)	124.9
**103**	5	–OC_2_H_5_	107.0	97.6	-	134.4
**104**	7	–CH_3_	63.9	-	74.0	111.5
**105**	9	–CH_3_	69.6	-	75.8	113.6
**106**	15	–CH_3_	60.5	60.3	-	100.3

**Table 12:** Transition temperatures (T_A-B_, °C) of symmetrical dimers containing an (R)-2-methylpentamethylene spacer.

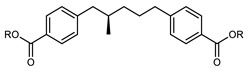					
**No.**	**R**	**T_MP_**	T_SmA-N_TB_*_	T_N_TB_-N*_	T_N*-Iso_
**107**	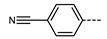	134.1	-	-	71.1
**108**	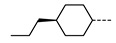	61.6	-	-	-
**109**	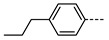	69.1	-	-	-
**110**	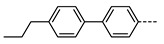	66.6	-	-	233.6
**111**	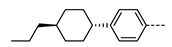	117.1	110.8	123.5	219.8
**112**	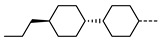	119.3	161.0	167.2	236.4
**113**	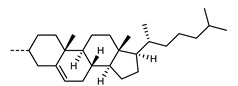	123.3	-	-	237.4

**Table 13:** Transition temperatures (T_A-B_, °C) of unsymmetrical CBnOPZPZP dimers with a terminal butyl, (S)-2-methylbutyl, or *rac* 2-methylbutyl chain [71].

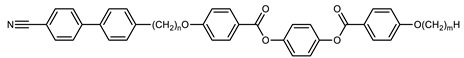						
**No**	***n***	***m***	**T_MP_/°C**	T_SmA-N_TB_*_	T_N_TB_-N*_	T_N*-Iso_
**114**	4	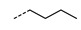	154	-	-	249
**115**	6	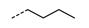	154	-	96	248
**116**	8	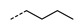	144	-	93	241
**117**	10	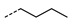	135	-	89	231
**118**	4	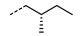	162	-	-	209
**119**	6	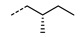	153	-	89	214
**120**	8	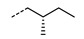	132	-	93	212
**121**	10	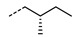	131	88	-	203
**122**	6	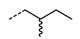	153	-	85	214
**123**	8	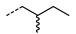	131	-	92	207

**Table 14:** Transition temperatures (T_A-B_, °C) of N-phenyl piperazine derived bent-core compounds [12,73].

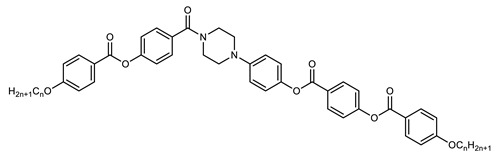						
**No.**	***n***	**T_MP_**	**T_SmC_P_-N/Iso_**	**T_Col_X_-N_**	**T_N_TB_-N_**	**T_N-Iso_**
**124**	4	201	-	-	193	212
**125**	5	187	-	-	172	192
**126**	6	176	-	157	169	188
**127**	7	169	-	-	-	186
**128**	8	177	180	-	-	180.5
**129**	9	167	185	-	-	-
**130**	10	162	191	-	-	-
**131**	11	161	194	-	-	-
**132**	12	165	201	-	-	-
**133**	16	142	193	-	-	-

**Table 15:** Transition temperatures (T_A-B_, °C) of the hydrogen-bonded trimers CB5OCB (linear) and CB6OBA (bent) [86].

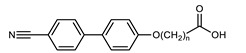					
**No.**	**Name**	***n***	**T_MP_**	**T_N_TB_-N_**	**T_N-Iso_**
**135**	*CB5OBA*	5	196	-	209
**136**	*CB6OCB*	6	160	159	197

**Table 16:** Transition temperatures (T_A-B_, °C) of compounds **140**–**143**, the ‘DTC5-C9’ family [92].

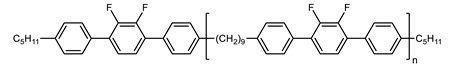					
**No.**	***n***	**T_MP_**	**T_SmX-N_**	**T_N_TB_-N_**	**T_N-Iso_**
**140**	0	34	-	-	116.5
**141**	1	77	85	124	162
**142**	2	127	-	145	192
**143**	3	142	-	168	205

**Table 17:** Transition temperatures (T_A-B_, °C) of ether-linked trimers **144**–**151** [99].

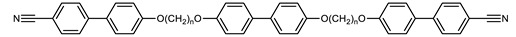					
**No.**	***n***	**T_MP_**	**T_SmA-N_**	**T_N_TB_-N_**	**T_N-Iso_**
**144**	4	230.1	197	-	297.4
**145**	5	176.6	-	122	215.4
**146**	6	217.4	192	-	257.8
**147**	7	162.9	-	132	207.2
**148**	8	201.0	175	-	231.9
**149**	9	155.0	-	135	195.8
**150**	10	198.0	150	-	210.8
**151**	11	147.4	-	130.5	185.9

**Table 18:** Transition temperatures (T_A-B_, °C) of mixed ether/thioether linked timers **152**–**160** [100].

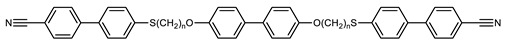					
**No.**	***n***	**T_MP_**	**T_SmA-N_**	**T_N_TB_-N_**	**T_N-Iso_**
**152**	3	186.4	-	-	142.0
**153**	4	235.3	-	-	253.2
**154**	5	171.8	-	117	154.2
**155**	6	219.0	180	-	217.4
**156**	7	149.4	-	126	162.4
**157**	8	195.6	167	-	198.8
**158**	9	131.3	-	122.7	158.8
**159**	10	184.8	146	-	176.2
**160**	11	135.4	-	121	154.4

**Table 19:** Transition temperatures (T_A-B_, °C) of compounds **161**–**164**; # sample decomposes at and above 230 °C [101].

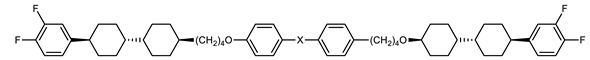				
**No.**	**X**	**T_MP_**	**T_N_TB_-N_**	**T_N-Iso_**
**161**	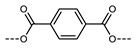	178.8	166.2	304.6
**162**	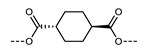	169.3	159.8	285.5
**163**	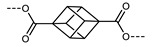	174.4	163.9	>230 ^#^
**164**	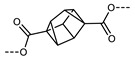	162.2	-	>230 ^#^

**Table 20:** Transition temperatures (T_A-B_, °C) of compounds **77**, **165**–**172**. *Link Seq.* refers to the shape (B = bent, L = linear) shape of the all *trans* conformation of the linkage units [102].

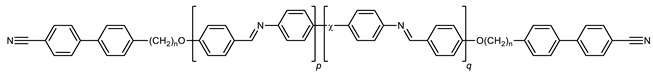								
**No.**	***n***	**χ**	***p***	***q***	**T_MP_**	**T_N_TB_-N_**	**T_N-Iso_**	**Link Seq.**
**77**	6	-	0	0	99	109	155	*B*
**165**	6	-	1	0	153	142	185	*BB*
**166**	6	–(CH_2_)_7_–	1	1	140	159	180	*BBB*
**167**	6	–(CH_2_)_6_O–	1	1	151	158	195	*BBB*
**168**	6	–O(CH_2_)_5_O–	1	1	156	162	215	*BBB*
**169**	6	–O(CH_2_)_6_O–	1	1	166	166	235	*BLB*
**170**	6	–(CH_2_)_8_–	1	1	131	152	212	*BLB*
**171**	7	–(CH_2_)_7_–	1	1	145	132	225	*LBL*
**172**	O6	–(CH_2_)_7_–	1	1	131	143	242	*LBL*

**Table 21:** Transition temperatures (T_A-B_, °C) of compound **173** and complexes **174** and **175** [103].

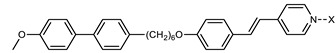						
**No.**	**X**	**T_MP_**	**T_SmX_**	**T_SmA_-_NTB_**	**T_N_TB_-N_**	**T_N-Iso_**
**173**	none	142.8	-	-	-	-
**174**	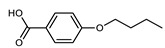	121.9	86.2	-	109.4	166.4
**175**	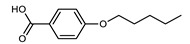	112.0	85.5	93.5	98.0	157.7

**Table 22:** Transition temperatures (T_A-B_, °C) of complexes **176** and **177** [105].

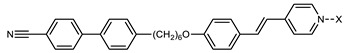				
**No.**	**X**	**T_MP_/°C**	**T_N_TB_-N_/°C**	**T_N-Iso_/°C**
**176**	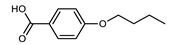	107	95	167
**177**	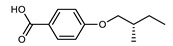	103	88	145

**Table 23:** Transition temperatures (T_A-B_, °C) of complexes **178**–**184** [106].

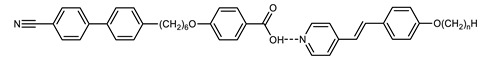					
**No.**	***n***	**T_MP_**	**T_SmC-N_TB__**	**T_N_TB_-N_**	**T_N-Iso_**
**178**	1	130	-	110	182
**179**	2	119	-	115	190
**180**	3	109	-	108	173
**181**	4	108	86	113	180
**182**	5	121	92	107	165
**183**	6	105	96	106	158
**184**	7	100	100	104	155

**Table 24:** Transition temperatures (T_A-B_, °C) of complexes **185** and **186** [106].

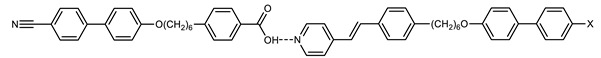				
**No.**	**X**	**T_MP_**	**T_N_TB_-N_**	**T_N-Iso_**
**185**	-CN	127	143	186
**186**	-OCH_3_	140	142	184
